# Dietary total antioxidant capacity and risk of breast cancer: a systematic review and dose-response meta-analysis of observational studies

**DOI:** 10.3389/fnut.2026.1890538

**Published:** 2026-08-07

**Authors:** Jianying Zhou, Haiyan Jin, Jing Shan, Ziqing Ye

**Affiliations:** Healthcare Center, Zhejiang Hospital, Hangzhou, Zhejiang Province, China

**Keywords:** breast cancer, dietary total antioxidant capacity, dose-response meta-analysis, observational studies, systematic review

## Abstract

**Background:**

Numerous epidemiological studies have suggested a potential association between the antioxidant capacity of the diet and the risk of breast cancer. Yet, these studies have yielded inconclusive results. We therefore conducted a systematic review and dose-response meta-analysis of observational studies to ascertain the association between dietary total antioxidant capacity (TAC) and breast cancer risk.

**Methods:**

We systematically searched PubMed, Embase, Scopus, ISI Web of Science and China National Knowledge Infrastructure (CNKI) from database inception to May 2026, using predefined keywords to identify the relevant articles. Random-effects or fixed-effects models were used to pool relative risks (RRs) with corresponding 95% confidence intervals (CIs), as appropriate. Heterogeneity across studies was estimated using Cochran's Q test and the I^2^ statistics.

**Results:**

Nine observational studies (seven case-control and two cohort studies), encompassing 33,981 participants, were included in the meta-analysis. Higher dietary TAC intake was significantly associated with a lower risk of breast cancer (RR = 0.86; 95%CI: 0.76–0.98; *P* = 0.022). Conversely, each standard deviation increment in dietary TAC intake was not associated with the risk of breast cancer (RR = 0.98; 95%CI: 0.94–1.02, *P* = 0.295). Also, the dose-response analysis showed a linear inverse association between dietary TAC and risk of breast cancer (RR = 0.988; 95%CI: 0.981–0.995, *P*_*dose*−*response*_=0.001, *P*_*nonlinearity*_ = 0.546). The results of subgroup analyses showed a significant inverse association between dietary TAC and breast cancer in cohort studies, with low heterogeneity (*P* = 0.174; *I*^2^ = 18.9%).

**Conclusions:**

This study indicates that higher dietary TAC intake is associated with a reduced risk of breast cancer. Further well-designed longitudinal studies are warranted to confirm these findings.

## Introduction

Breast cancer is the most commonly diagnosed cancer and the leading cause of malignancy-related deaths in females (1). According to GLOBOCAN 2020, breast cancer has surpassed lung cancer to become the most commonly diagnosed cancer worldwide, with an estimated approximately 2.3 million new cases and 685,000 deaths (2). In China, breast cancer is an increasingly serious health challenge, with approximately 357, 200 cases and 75,000 deaths of female breast cancer occurring in 2022 (3). As the global incidence of this disease continues to rise, identification of risk factors is of paramount importance for developing effective preventive strategies. The well-established risk factors for breast cancer included genetic predisposition, race, family history of breast cancer, use of exogenous hormones, reproductive history, increased alcohol consumption, smoking, obesity, physical inactivity (4). Apart from the above-mentioned these factors, diet, as a modifiable risk factor, has also long been perceived to play a prominent role in the prevention of breast cancer (5).

Over recent decades, a large volume of epidemiological studies have examined the associations between the intakes of certain nutrients, foods, whole dietary patterns and risk of breast cancer (5, 6). Of concern, a previous systematic review and meta-analysis found that high consumption of fruits, vegetables and soy isoflavone were significantly associated with reduced risk of breast cancer (7). The authors speculate that fruits and vegetables are abundant in antioxidants, which may help to reduce the risk of breast cancer by inhibiting oxidative stress and inflammation (8). Actually, the majority of previous studies have primarily focused on the impacts of intake of specific antioxidant nutrients or nutrient groups on breast cancer risk (9–11). For example, a recently published systematic review and meta-analysis showed a significant inverse association between vitamin C and risk of breast cancer (11). Nonetheless, less research attention to date has been given to the impact of antioxidant capacity of the diet on breast cancer. Simultaneously, single antioxidant may not fully reflect the total antioxidant capacity of the diet (12), due to the complexity of diet and the potential synergistic effects of antioxidants. Consequently, the concept of dietary total antioxidant capacity (TAC) has been proposed as a useful tool for evaluating the diet's total antioxidant potential, considering the cumulative and synergistic abilities of antioxidants present in mixed diets (13).

More recently, the potential role of dietary TAC has aroused growing concern in scientific research (14). A growing body of epidemiological evidence suggests that dietary high TAC intake is inversely associated with a range of health outcomes, including hypertension, sarcopenia, ulcerative colitis, depression and all-cause mortality (15–18). Notably, the findings of a recent systematic review and dose-response meta-analysis by Li and colleagues indicated that higher dietary TAC intake was inversely associated with the risk of prediabetes and diabetes mellitus (19). However, existing epidemiological evidence relating dietary TAC to breast cancer remains limited. As of now, only ten observational studies have explored this topic, yielding inconsistent results (20–29). Some studies have demonstrated an inverse association between dietary TAC and breast cancer (20, 23, 27, 28), whereas other studies showed no statistically significant association (21, 24–26, 29). Notably, a previous systematic review and meta-analysis of 21 studies found that higher intake of dietary TAC was associated with a reduced risk of breast cancer in meta-analysis of prospective studies (30). However, this meta-analysis only included three observational studies (2 cohort and 1 case-control studies) and systematically searched for relevant studies published up to February 2020. Furthermore, several new studies have been published since 2020 (21, 23, 27–29). Therefore, to clarify the overall association between high dietary TAC intake and breast cancer risk, we undertook the current systematic review and dose-response meta-analysis to synthesize the available findings from observational studies published up to May, 2026.

## Methods

### Search strategy

This study was performed according to the Preferred Reporting Items for Systematic Reviews and Meta-analyses (PRISMA) guidelines (31). A systematic search of relevant studies published up to May 2026, was performed in the PubMed, Web of Science, Embase, Scopus and CNKI databases through examining the following combination of keywords: “dietary total antioxidant capacity”, “dietary TAC”, “dietary antioxidant capacity”, “non enzymatic antioxidant capacity”, “dietary antioxidant index”, “antioxidant capacity of diet” and “breast cancer”, “breast tumor”, “breast adenoma”, “breast neoplasms”, “breast carcinoma”. The search was limited to human studies with no restrictions in terms of publication date or language. In addition to the initial database search, hand-searching from reference lists of all selected articles and reviews was also performed to find potentially relevant articles. Notably, gray literature or unpublished studies were not eligible in this meta-analysis. Detailed information about search strategy of each database can be found in Supplementary Table 1.

### Study selection

Two of the authors (J.-Y.Z and H.-Y. J) independently screened the titles and abstracts of all potential articles retrieved in the initial literature search, and removed duplicates and irrelevant articles. Subsequently, the full-text versions of the remaining articles were reviewed according to the criteria for inclusion and exclusion. Eligible studies were included in this study, if they met all of eligibility criteria: (1) observational study, e.g. cohort, case-control or cross-sectional study;(2) performed in adults aged ≥18 years;(3) reported the main exposure of interest as dietary TAC; (4) reported the outcome of interest as breast cancer; (5) provided the multivariable adjusted risk estimates (ORs, HRs, or RRs) and corresponding 95% confidence intervals (CIs) (or sufficient information to calculate them); (6) If the retrieved article lacked sufficient details, the corresponding author would be contacted by email. In addition, the criteria for exclusion were as follows: (1) non-observational studies, such as reviews, books, editorials and conference paper; (2) did not provide the risk estimates and corresponding 95% CIs;(3) the exposure of interest was individual antioxidant intake, such as vitamin C and β-Carotene; (4) Irrelevant articles. Any disagreements about the inclusion and exclusion of eligible studies were addressed by consensus or by the corresponding author (Z.-Q.Y.) if need. The study population, exposure, comparator, outcome, and study design (PECOS) information is illustrated in Table 1.

**Table 1:** The PECOS criteria used for this systematic review and dose-response meta-analysis.

Population	Adults
Exposure	Dietary total antioxidant capacity
Comparator	Highest category vs. lowest category of exposure
Outcomes	Breast cancer
Study design	Observational studies including cohort, case-control or cross-sectional designs

PECOS, population, exposure, comparator, outcome and study design.

### Data extraction

Two authors (J.-Y.Z and J.S.) independently extracted the following data from each eligible article, including first author's name, publication year, study design, study region, sample size, numbers of total participants and breast cancer cases, mean age/age range, follow-up duration for cohort studies, dietary assessment methods, confounding variables that were adjusted for in the multivariate analyses, and reported risk estimates with their corresponding 95% CIs of breast cancer across different categories of dietary TAC.

### Quality assessment

Each selected study was assessed for methodological quality using the Newcastle-Ottawa Scale (NOS) by the two aforementioned authors (H.-Y.J and J.S.), with any discrepancies resolved by consensus or corresponding author (Z.-Q.Y.). This NOS tool contains eight items in three domains: selection of participants, comparability of participants, and ascertainment of outcome/exposure of interest, which is a validated scale for non-randomized studies in meta-analyses (32). Only those studies with total NOS scores ≥7 points were recognized as high quality (33).

### Definition of dietary TAC

Dietary TAC, also known as non-enzymatic antioxidant capacity, is developed as a unique and suitable tool for evaluating the total dietary antioxidant capability from foods and beverages (34). Several different chemical assays are used to evaluate dietary TAC, such as the ferric reducing antioxidant potential (FRAP), vitamin C equivalent antioxidant capacity (VCEAC), trolox equivalent antioxidant capacity (TEAC), oxygen radical absorbance capacity (ORAC), and total radical-trapping antioxidant parameter (TRAP) (30). The unit of dietary TAC was defined as millimoles of Trolox equivalents per day (mmol TE/day), calculated using the FRAP or ORAC assays, depending on the dietary database used in the original studies.

### Data synthesis and statistical analyses

In this systematic review and meta analysis, RRs and their 95% CIs were regarded as the main risk estimate for all eligible studies. Moreover, we considered that HR was approximately equal to RR (35). For the cross-sectional and case-control studies, OR was converted into RR using the following formula: RR=OR/ [(1-P_0_) + (${\text{P}}_{0}^{*}$OR)], where P_0_ shows the incidence of breast cancer in the non-exposed group (36). We extracted this information from each original study or estimated it using the reported overall incidence. A pairwise meta-analysis was performed by pooling the RRs and 95% CIs of breast cancer associated with the highest vs. lowest categories of dietary TAC. Inter-study heterogeneity was assessed using the Cochran's *Q*-test and *I*^2^ statistics (37), with a *P*-value of *Q*-test < 0.10 or *I*^2^ > 50% considered to be substantial heterogeneity. Because of anticipated heterogeneity among the included studies, the pooled RRs were calculated using a random-effects model (DerSimonian and Laird methods) (37). To further identify the possible sources of heterogeneity across all eligible studies and check the robustness of observed association, we performed sensitivity and subgroup analyses. In our analyses, subgroup analyses were conducted to explore potential effects attributable to variables, including study design (cohort or case-control studies), study region (Western or Asian countries), study quality (≥7 or < 7), mean age (≥50y or < 50y), case size (< 1,000 or ≥1,000), menopausal status (premenopause or postmenopause) and methods for dietary TAC (FRAP/ORAC/TEAC or DAI). Publication bias was visualized using the funnel plots and quantified by both Begg's and Egger's regression asymmetry tests (38). If the results showed evidence of publication bias, the trim and fill method was used to recalculate the combined RRs (39). Meanwhile, we also performed a dose-response meta-analysis to estimate the trend of the relevant log RRs across the categories of dietary TAC scores. TAC values were standardized to mmol TE/day. A two-stage GLST model based on generalized least square method was adopted to investigate the linear or non-linear dose-response association between dietary TAC and breast cancer (40). All statistical tests were two-tailed, and used a significance level of *P*-value < 0.05. We used STATA 16.0 (Stata Corp, College Station, TX, USA) for all statistical analyses in this study.

## Results

### Search results

**Figure 1:**
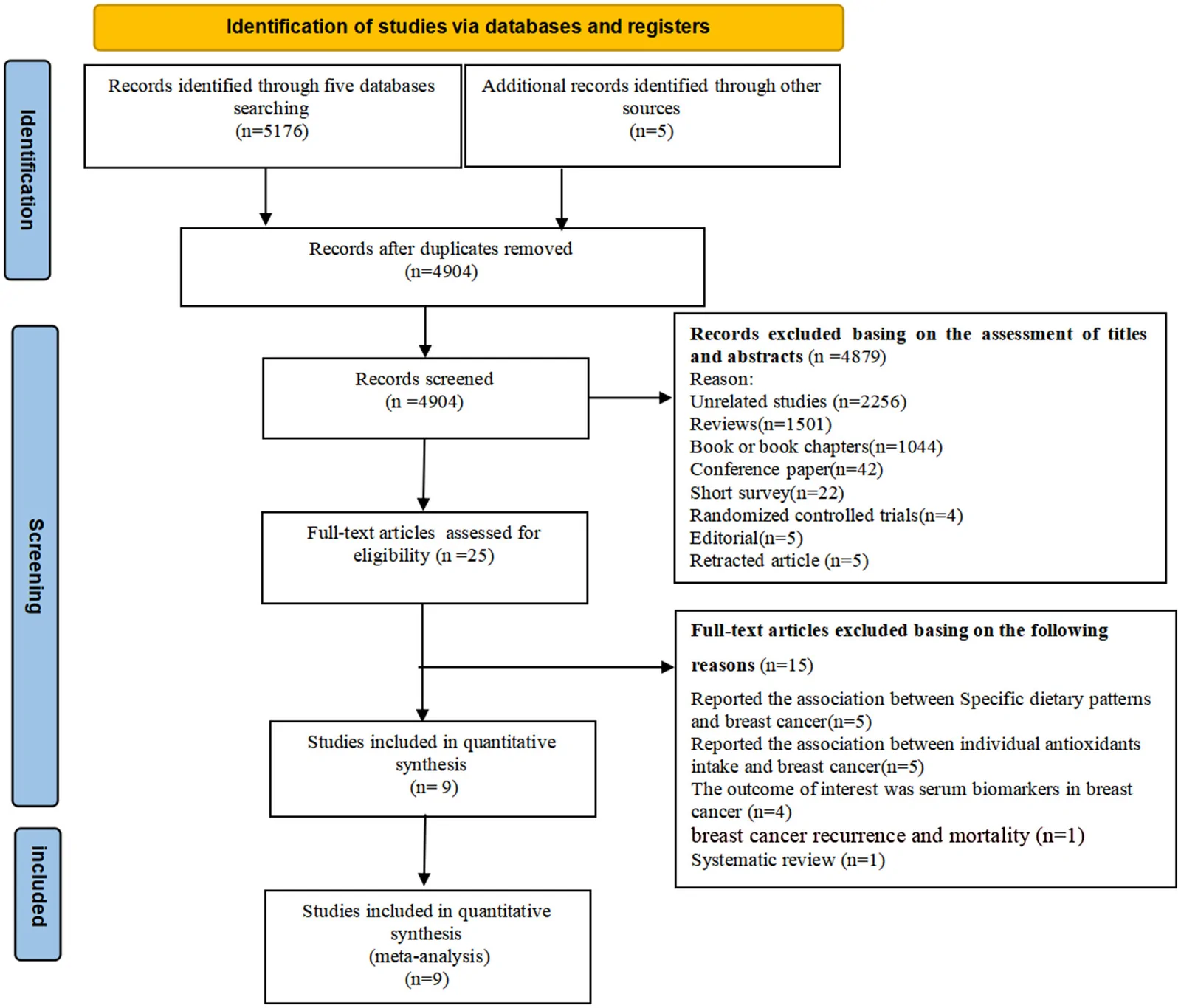
Flow chart of the process of the study selection.

The flowchart regarding literature search process is shown in Figure 1. Our initial literature search yielded a total of 5,181 potential articles, and after removing duplicates, 4,904 articles were considered for further screening. Subsequently, 4,879 articles were excluded based on the assessment of titles and abstracts of potential articles and the full-text versions of the remaining 25 articles were carefully checked for eligibility. Out of the remaining 25 articles, 16 articles were excluded based on the following reasons: reported the association between specific dietary patterns and breast cancer (*n* = 5), reported the association between individual antioxidants intake and breast cancer (*n* = 5), outcomes of interest was biomarkers in breast cancer (*n* = 4), breast cancer recurrence and mortality (*n* = 1), and systematic review (*n* = 1). Finally, nine articles met the eligibility criteria and were included in our final analysis.

### Characteristics of the included studies

Descriptive characteristics of 10 observational studies included in the meta-analysis are shown in Table 2. Seven case-control (21, 23–28) and two cohort studies (20, 29) were included in this study. All included studies were published between 2015 and 2025. The included studies were performed across four different countries, including Iran (21, 23, 24, 26–28), Netherlands (20), Spain (25) and Sweden (29). The age of participants among included studies ranged from ages 18 to above. Sample sizes of included studies ranged from 275 to 24,950. The follow-up duration for the cohort studies ranged from 17 to 19.2 years. For dietary assessment methods, all the included studies used FFQs to collect dietary data (20, 21, 23–29). To measure the quantitative value of dietary TAC, four studies used the FRAP assay (20, 21, 23, 29), three studies used the ORAC assay (21, 24, 26), two studies used DAI (27, 28), and one study used the TEAC assay (25). Based on the NOS, seven studies were classified as high quality (20, 23, 25–29), and the remaining two studies were classified as medium quality (21, 24). The quality assessment of included studies bases on NOS criteria is shown in Table 3.

**Table 2:** Characteristics of the included studies on the association between dietary total antioxidant capacity and breast cancer risk.

References	Study region	Study design	Total number of participants	Age	Exposure assessment	Adjustment or matched for in the analyses	Outcomes
Pantavos et al. (20)	Netherlands	Cohort	3,209 (199 cases)	≥55y	FFQ	Age, BMI, educational level, family history of breast cancer (yes or no), smoking status (never or former and current) and alcohol consumption, use of multivitamin supplement (yes or no)	Tertile 3 vs Tertile 1 of TAC (HR= 0.68, 95% CI:0.49-0.96)
Jalali et al. (21)	Iran	Case-control	136 cases 272 controls	≥30y	FFQ	Age, first pregnancy age, menopausal status, energy.	Highest quartile 4 vs lowest quartile 1 of TAC (OR = 1.04,95% CI: 0.53-2.05).
Sadanfar et al. (23)	Iran	Case-control	412 cases 456 controls	19-80y	FFQ	Age, energy, physical activity, family history of breast cancer, menopausal hormone use, education, parity, oral contraceptive use, cigar smoking, alcohol consumption, fertility treatment, marital status, folic acid, B6 and BMI.	Highest quartile 4 vs lowest quartile 1 of TAC (OR = 0.61,95% CI: 0.38-0.99).
Karimi et al. (24)	Iran	Case-control	100 cases 175 controls	30-65y	FFQ	Age (years), age at menarche (years), age at first pregnancy (years), number of full pregnancies, smoking(yes/no), use of oral contraceptives (yes/no), use of brassiere per day (< 12 h/>12 h), body mass index (kg/m^2^), life satisfaction (yes/no/partly), menopause status (yes/no), family history of breast cancer (yes/no), physical activity (METs h/week), energy intake (kcal/day), and energy density of the diet (kcal/100 g of foods).	Highest quartile 4 vs lowest quartile 1 of TAC (OR = 0.43,95% CI: 0.11-1.12).
Obón-Santacana et al. (25)	Spain	Case-control	1486 cases 1652 controls	≥18y	FFQ	Age, study area, educational level, family history of breast cancer, tobacco smoking, HRT use, OC use, age at menarche, age at first pregnancy, number of children, menopausal status, physical activity, and BMI.	Highest quartile 4 vs lowest quartile 1 of TAC (OR = 1.09,95% CI: 0.90-1.32).
Safabakhsh et al. (26)	Iran	Case-control	150 cases 150 controls	24-73y	FFQ	Age (year), BMI (kg/m^2^), physical activity (MET/h/wk), education ( ≤ high school or>university degree), marital status (married or single/divorced/widowed), socioeconomic status (low or high/average), alcohol use (yes or no), smoking (yes or no), vitamin supplements (yes or no) and medication (lipid lowering and anti-hypertensive medications) use (yes or no), comorbidities (diabetes, hypertension and hyperlipidemia) (yes or no), length of oral contraceptives (OCP) use (year), HRT(yes or no), systolic BP, diastolic BP (mm/Hg), age at menarche (year), time since menopause in post-menopausal women (year), weight at age 18 years old (kg), number of child (n), breast feeding ages (year), family history of BrCa (yes or no), dietary intake of fiber (g), tea (g), coffee (g) and total energy (kcal/day).	Highest tertile 3 vs lowest tertile 1 of TAC(RR = 0.77,95% CI: 0.23-4.26).
Vahid et al. (27)	Iran	Case-control	145 cases 148 controls	≥18y	FFQ	Age, education, BMI, occupation, alcohol consumption, smoking, pregnancy, family history, menarche age, MET, HRT, and total calorie intake.	Highest vs lowest intake of TAC (OR = 0.18,95% CI: 0.09-0.37).
Allahyaru et al. (28)	Iran	Case-control	180 cases 360 controls	35-65y	FFQ	Age, BMI, number of pregnancies, number of abortions, breastfeeding duration, age of menopause, and total calorie intake	Highest vs lowest intake of TAC (OR = 0.93,95% CI: 0.89-0.96).
Mariosa et al. (29)	Sweden	Cohort	24,950 (1142 cases)	≥18y	FFQ	Age, body mass index, menopausal status, energy intake, educational level, cigarette smoking status, alcohol drinking, coffee drinking, physical activity, vitamins and minerals use, contraceptive pill use, hormone replacement therapy, age at the first menstruation, number of children and childlessness.	Highest quartile 4 vs lowest quartile 1 of TAC (HR = 0.85,95% CI: 0.69-1.04).

BMI, body mass index; CI, confidence interval; FFQ, Food frequency questionnaire; HR, hazard ratio; HRT, hormone replacement therapy; OR, odds ratio; TAC, total antioxidant capacity.

**Table 3:** Dietary total antioxidant capacity and risk of breast cancer: assessment of study quality.

References	Selection				Comparability		Outcome			Score
	1	2	3	4	5A	5B	6	7	8	
Case-control										
Jalali et al. (21)	^*^	^*^	^*^		^*^		^*^	^*^		6
Sadanfar et al. (23)	^*^	^*^	^*^	^*^	^*^		^*^	^*^		7
Karimi et al. (24)	^*^	^*^	^*^		^*^		^*^	^*^		6
Obón-Santacana et al. (25)	^*^	^*^	^*^		^*^	^*^	^*^	^*^		7
Safabakhsh et al. (26)	^*^	^*^	^*^	^*^	^*^		^*^	^*^		7
Vahid et al. (27)	^*^	^*^	^*^	^*^	^*^		^*^	^*^	^*^	8
Allahyaru et al. (28)	^*^	^*^	^*^		^*^	^*^	^*^	^*^		7
Cohort										
Pantavos et al. (20)	^*^	^*^	^*^	^*^	^*^	^*^	^*^	^*^	^*^	9
Mariosa et al. (29)	^*^	^*^	^*^	^*^	^*^	^*^	^*^	^*^	^*^	9

^*^For case-control studies, 1 indicates cases independently validated; 2, cases are representative of population; 3, community controls; 4, controls have no history of breast cancer; 5A, study controls for the most important factor; 5B, study controls for additional factor(s), e.g. cigarette smoking body mass index, total energy intake; 6, ascertainment of exposure by blinded interview or record; 7, same method of ascertainment used for cases and controls; and 8, non response rate the same for cases and controls. For cohort studies, 1 indicates exposed cohort truly representative; 2, non exposed cohort drawn from the same community; 3, ascertainment of exposure by secure record(e.g. surgical records) or structured interview; 4, outcome of interest was not present at start of study; 5A, study controls for the most important factor; 5B, study controls for additional factor(s); 6, assessment of outcome is based on independent blind assessment or record linkage; 7, follow-up long enough(≥5 years) for outcomes to occur; and 8, adequacy of follow up of cohorts(all participants complete follow up or >90% participants complete follow up).

### Dietary TAC and breast cancer risk

**Figure 2:**
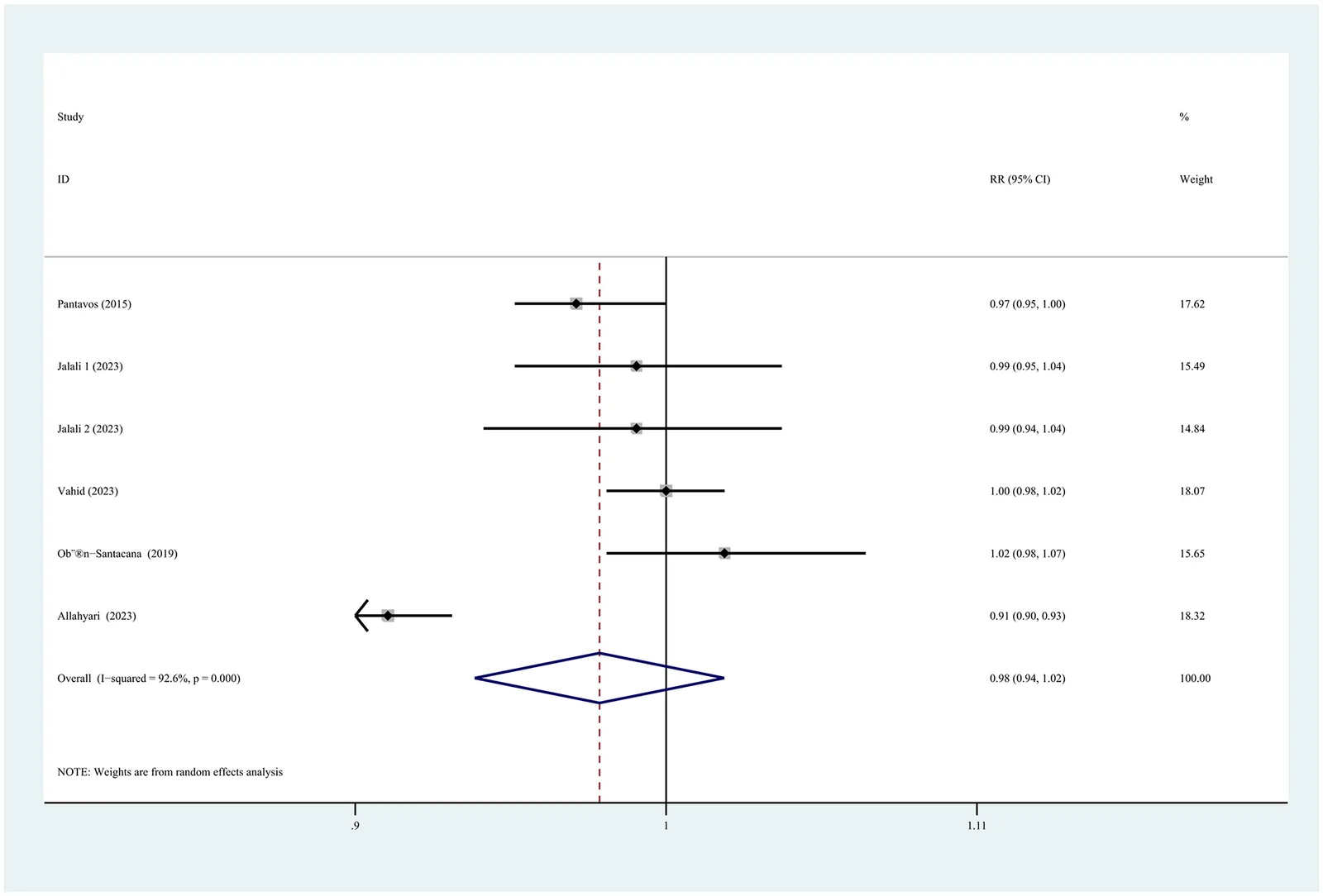
Forest plot of the association between dietary TAC intake and risk of breast cancer.

**Figure 3:**
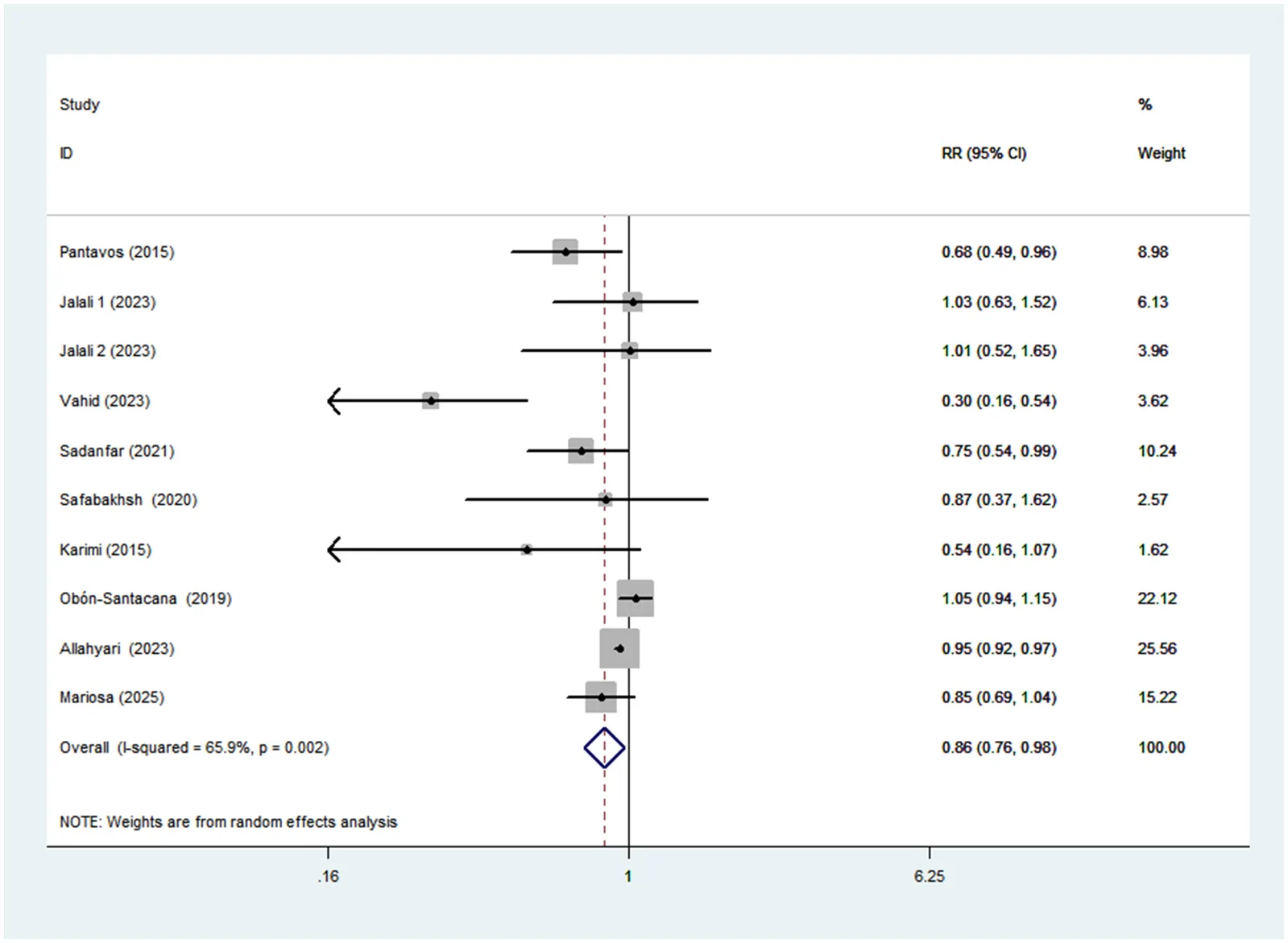
Forest plot of the association between each 1 SD increment in dietary TAC intake and risk of breast cancer.

Nine studies involving 33,981 participants and 3,950 cases, were included to assess the association between dietary TAC intake and breast cancer risk. Combining 10 effect sizes from nine studies, Figure 2 showed that higher dietary TAC intake was associated with a reduced risk of breast cancer (RR = 0.86; 95%CI: 0.76–0.98; *P* = 0.022), with evidence of substantial heterogeneity (I^2^ = 65.9%, *P* = 0.002). Thus, a random-effects model was used to pool the effect size. Additionally, Figure 3 showed that each SD increment in dietary TAC intake was not associated with the risk of breast cancer (RR = 0.98; 95%CI: 0.94–1.02, *P* = 0.295; *I*^2^ = 92.6%; *P* = 0.000).

### Dose-response analysis

**Figure 4:**
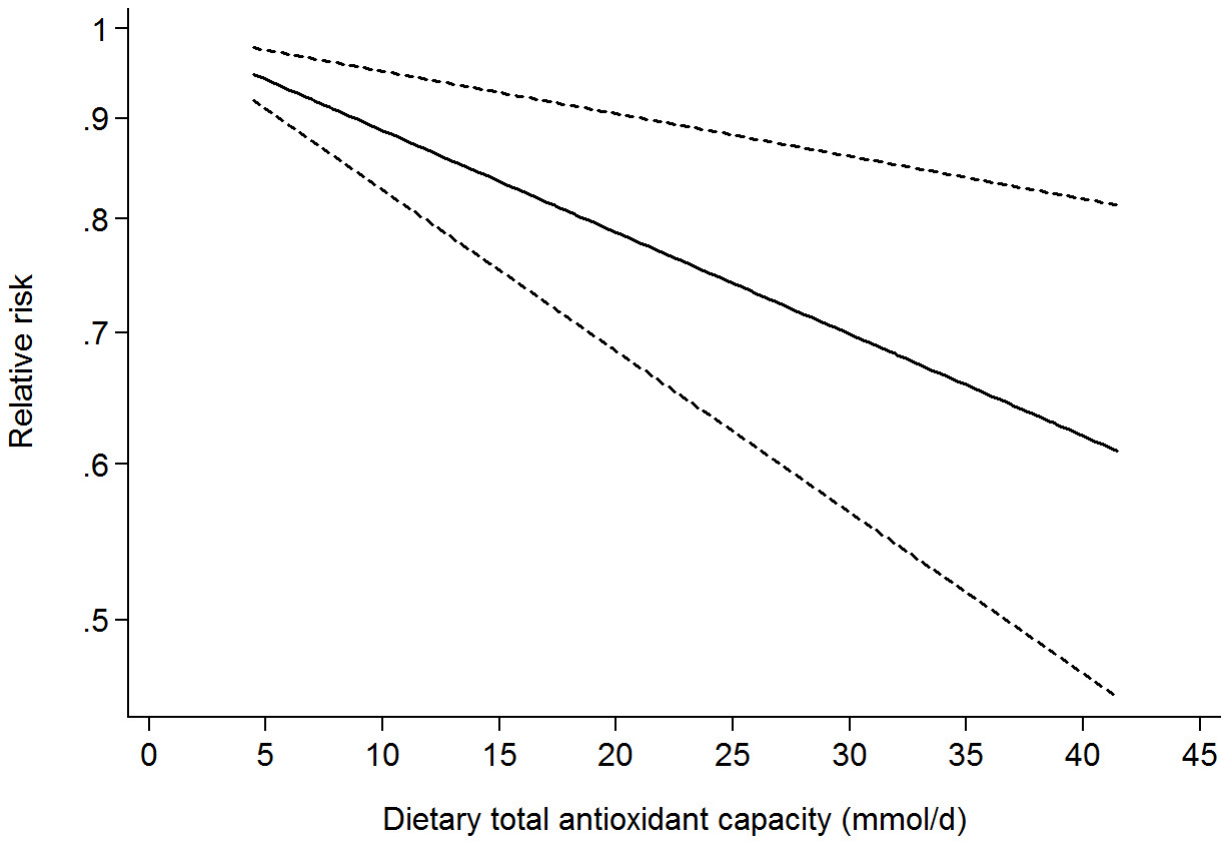
Dose-response analysis for the association between dietary TAC intake and risk of breast cancer.

As shown in Figure 4, the dose-response analysis showed a linear inverse association between dietary TAC and risk of breast cancer. The pooled RR was 0.988 (95%CI: 0.981–0.995) per 1 mmol TE/day increase (*P*_*dose*−*response*_ = 0.001, *P*_*nonlinearity*_ = 0.546).

### Results of subgroup analyses and meta-regression

To further clarify the potential sources of heterogeneity among the included studies, we carried out subgroup analyses stratified by study design, study region, study quality, menopausal status, mean age, case size, and methods for dietary TAC (Table 4). The results of subgroup analyses showed a significant inverse association between dietary TAC and breast cancer in cohort studies (RR = 0.79, 95%CI: 0.65–0.97, *P* = 0.025), and there was low heterogeneity (*P* = 0.174; *I*^2^ = 18.9%). Additionally, the results showed no statistical association between dietary TAC and breast cancer in the subgroup with study quality < 7 and premenopausal women, but there was no evidence of heterogeneity (*I*^2^ = 0.0%). Meta-regression analysis showed that study design and participants' menopausal status explained 47.0%, 65.9% of the total variance across the studies, respectively.

**Table 4:** Subgroup analyses of breast cancer for the highest vs. lowest category of dietary total antioxidant capacity intake.

Dietary total antioxidant capacity	Subgroup	No. of studies	RR(95%CI)	*P*-values	Heterogeneity		
					*P*-values for within groups	*I*^2^(%)	*P-*values for between groups
Study design	Cohort	2	0.79 (0.65–0.97)	0.025	0.174	18.9	0.054
	Case-control	7	0.89 (0.77–1.03)	0.117	0.003	67.4	
Study region	Western countries	3	0.88 (0.70–1.11)	0.293	0.017	75.6	0.433
	Asian countries	6	0.78 (0.60–1.01)	0.062	0.007	66.0	
Study quality	≥7	7	0.85 (0.74–0.98)	0.021	0.000	75.9	0.992
	< 7	2	0.95 (0.68–1.32)	0.747	0.465	0.0	
Menopausal status	Premenopause	5	0.94 (0.83–1.06)	0.326	0.575	0.0	0.333
	Postmenopause	5	0.95 (073–1.25)	0.738	0.012	66.0	
Mean age	≥50y	5	0.94 (0.86–1.03)	0.208	0.068	54.2	0.019
	< 50y	4	0.69 (0.46–1.07)	0.079	0.016	61.7	
Case size	≥1,000	2	0.96 (0.79–1.18)	0.720	0.070	69.5	0.176
	< 1,000	7	0.77 (0.61–0.97)	0.024	0.003	67.2	
Methods for dietary TAC	FRAP/ORAC/TEAC	7	0.88 (0.76–1.02)	0.098	0.083	44.4	0.784
	DAI	2	0.56 (0.18–1.72)	0.308	0.000	92.7	

CI, confidence interval; FFQ, Food frequency questionnaire; RR, relative risk.

### Publication bias

As shown in Supplementary Figure 1, inspection of funnel plots revealed little evidence of asymmetry. Moreover, no evidence of publication bias was observed based on Begg's and Egger's tests (highest compared with lowest categories of dietary TAC: Begg's test: *P* = 0.210; Egger's test: *P* = 0.133).

### Sensitivity analysis

As shown in Supplementary Figure 2, Allahyari et al.' study may be the potential source of heterogeneity. After removing studies of Obón-Santacana et al., and Allahyari et al. in the repeat analysis (Supplementary Figure 3), the results showed a significant decrease in the pooled RRs on the association between dietary TAC and breast cancer risk (RR = 0.75; 95%CI: 0.60–0.93, *P* = 0.008). Meanwhile, the heterogeneity also decreased from 65.9 to 49.3%.

## Discussion

As far as we aware, this is the largest and most comprehensive systematic review and dose-response meta-analysis to date evaluating the relationship between dietary TAC and breast cancer risk. In this systematic review and meta-analysis of nine observational studies comprising 33,981 participants, we found that higher dietary TAC intake was significantly associated with a lower risk of breast cancer. In addition, the dose-response analysis showed a linear inverse association between dietary TAC and breast cancer risk. Collectively, our findings corroborate that there is an inverse association between higher dietary TAC intake and breast cancer risk.

Globally, breast cancer has now surpassed lung cancer as the leading cause of cancer incidence in 2020, with approximately 2.3 million new cases (2). Furthermore, due to population growth and the adoption of western lifestyles, the number of breast cancer cases in many developing countries is increasing (41). This upward trends reflects the urgency of taking effective measures to prevent breast cancer. Currently, there is relatively little knowledge regarding the effect of dietary TAC on breast cancer. Meanwhile, increasing epidemiological studies reported the association between dietary TAC intake and other types of cancers. Toorang et al. observed that dietary TAC could decrease the risk of head and neck cancer (OR = 0.49; 95%CI: 0.39–0.62) (42). Similarly, a previous systematic review and meta-analysis on dietary TAC and site-specific cancers found higher intake of dietary TAC was associated with 21% reduced risk of colorectal cancer, 27% reduced risk of endometrial cancer, 42% reduced risk of gastric cancer, and 32% reduced risk of pancreatic cancer (30). Up to date, only nine observational studies have assessed the association between dietary TAC and risk of breast cancer (20, 21, 23–29), but these results are not entirely consistent. For example, in the Rotterdam study, Pantavos et al., found that high dietary antioxidant capacity was associated with a lower risk of breast cancer (20). Consistently, Sadanfar and colleagues also found that high dietary TAC was inversely associated with risk of breast cancer among the whole population and postmenopausal women (23). In contrast, Mariosa et al. in the Swedish National March Cohort, found no significant association between high dietary TAC and breast caner risk (29). The inconsistency in results across diverse studies could be attributable to the following reasons. First, variability in the methods used to measure dietary TAC across studies could be the reason for varying results. Three studies used the ORAC assay to measure dietary TAC (21, 24, 26), four studies used the FRAP assay (20, 21, 23, 29), two studies used DAI (27, 28), and one study used the TEAC assay (25). FRAP mainly measures hydrophilic antioxidants, including vitamin C, glutathione, and polyphenols, and does not detect thiol-containing antioxidants. ORAC, by contrast, scavenges peroxyl radicals, and thus detects both hydrophilic and lipophilic compounds. TEAC is broadly applicable but relies on a non-physiological radical. DAI is composite score with variable weighting algorithms. Consequently, the same dietary intake may yield different TAC values depending on the assay used (30). Second, regional variation in dietary habits could be partially contribute to the discrepant results. Among all included studies, six studies were performed in Iran (21, 23, 24, 26–28), one in Netherlands (20), one in Korea (22), one in Spain (25), and one in Sweden (29). As is known to all, Iran, as a Middle Eastern country, has different dietary and cultural habits from other countries, such as Korea. Third, the types of food that contribute the most to dietary TAC vary greatly among different countries and regions. For example, in hospital-based case-control studies, Safabakhsh and colleagues reported that the main food group's contributors to DTAC were tea, vegetables, fruits, and juices (26). Fourth, seven of included studies are case-control studies in this meta-analysis. Notably, the inverse association was more pronounced in cohort studies (RR = 0.79; 95%CI: 0.65–0.97) than in case-control studies (RR = 0.89; 95% CI: 0.77–1.03). Case-control studies, particularly those performed in hospital are prone to recall bias, because dietary assessment is conducted after the diagnosis of breast cancer, which may lead to a misclassification of TAC intake between case and control groups. Moreover, case-control study is also prone to selection bias. In contrast, cohort studies prospectively assess dietary exposure before the onset of breast cancer, thus are less affected by recall and selection biases. However, cohort studies may still be affected by loss to follow-up and residual confounding due to imprecise measurement of covariates. Given these design-specific biases, the estimated value (RR = 0.79) derived from two cohort studies, may provide a more reliable estimate of the true association. To sum up, variations in methods for measuring dietary TAC, dietary habits across countries and regions, and types of foods may attribute to the discrepancies in the study results.

Albeit evidence regarding the relationship between dietary TAC and breast cancer risk is inconsistent, several biologically plausible mechanisms support the inverse association between dietary TAC and breast cancer risk. First, existing evidence indicates that antioxidants can neutralize reactive oxygen species and prevent damage from free radicals in carcinogens (43). Second, studies have demonstrated that high intake of dietary TAC is associated with a lower plasma concentration of high-sensitivity C-reactive protein (44). Available scientific evidence indicates that increased inflammation can damage insulin secretion, thereby leading to insulin resistance (45), known risk factor for breast cancer (46). Third, as discussed previously, coffee is the main contributor to dietary TAC in different countries and regions. Previous studies have shown an inverse association between coffee consumption and risk of breast cancer (47). Considering above, these mechanisms may explain the apparent effect of high dietary TAC intake on breast cancer.

In our analyses, each SD increment in dietary TAC intake was not associated with the risk of breast cancer (RR = 0.98; 95%CI: 0.94–1.02, *P* = 0.295; *I*^2^ = 92.6%; *P* = 0.000). In contrast, compared with lowest intake, highest intake of dietary TAC was significantly associated with a lower risk of breast cancer (RR = 0.86; 95%CI: 0.76–0.98; *P* = 0.022). The apparent discrepancy among different analytical approaches might be attributed to the following reasons. First, dietary TAC was estimated using different assays (e.g., FRAP, ORAC) and food composition databases across the included studies. As a result, the absolute TAC values and the corresponding SD vary considerably across studies. Therefore, per SD estimate (RR = 0.98) is not directly comparable to the per unit dose-response slope (RR = 0.988), as the latter is based on a standardized scale derived from the pooled dose-response curves, while the former is study-specific and influenced by each population's dietary distribution. Second, the SD of TAC intake varies basing on the differences in dietary habits, geographical regions, and dietary assessment methods. For instance, populations with homogeneous dietary habits (e.g., Iranian population) tend to have smaller SD, which may dilute the observed association. Conversely, the categorical analysis (highest vs. lowest) intentionally contrasts the extremes of the distribution, thereby maximizing the disparity in exposure levels and increasing the power to detect true association. This may explain why the categorical analysis (RR = 0.84) yielded a stronger effect estimate than per SD analysis (RR = 0.98).

In this study, the dose-response analysis revealed a linear inverse association between dietary TAC and risk of breast cancer (RR = 0.988; 95%CI: 0.981–0.995, *P*_*dose*−*response*_ = 0.001, *P*_*nonlinearity*_ = 0.546). This slope should be interpreted as a population-level trend rather than a clinically actionable threshold for individuals. A meaningful risk reduction is more likely achieved through a cumulative increase in TAC derived from a diverse and high-quality dietary pattern, rather than small daily increments. Although a 1.2% risk reduction per 1 mmol TE/day increase in dietary TAC was statistically significant, its clinical significance at the individual level should be interpreted cautiously. At the population level, however, even a small reduction in RR may translate into a considerable number of preventable cases. Collectively, our findings underscore the importance of cumulative high dietary antioxidant intake rather than marginal daily increment, in reducing the risk of breast cancer.

In this meta-analysis, we also found substantial heterogeneity for high dietary TAC intake and risk of breast cancer (*I*^2^ = 65.9%). Thus, the pooled estimates should be interpreted with appropriate caution. However, we believe that pooling remains justifiable for the following reasons: (1) All included studies were clinically homogeneous in terms of exposure (dietary TAC assessed by validated FFQs) and outcome (pathologically confirmed breast cancer); (2) We used random-effects models to incorporate between-study variance; and our subgroup analyses identified study design and menopausal status as significant contributors, explaining approximately 47.0%, 65.9% of the heterogeneity. However, significant heterogeneity remained in other subgroup analyses, indicating the presence of other unmeasured or unknown confounding factors. Further longitudinal studies or randomized controlled trials are warranted to confirm the association between high intake of dietary TAC and breast cancer risk. Furthermore, the extremely high heterogeneity in the SD increment analysis (*I*^2^ = 92.6%) suggests that per SD metric may not be suitable for pooling across studies due to population-specific variability in TAC distributions. Therefore, our primary conclusions are based on the categorical and dose-response analyses, while per SD results are interpreted as supportive but less reliable.

### Strengths and limitations

There are several notable strengths of this study. First, to the authors' knowledge, this is the latest and most comprehensive systematic review and dose-response meta-analysis discussing the association between dietary TAC and breast cancer risk. Compared with the previous meta-analyses (30), we not only provided timely update, but also included more original articles involving more participants and breast cancer cases, thereby providing more robust evidence. Also, we also performed a dose-response analysis to strengthen the correlation between dietary TAC and breast cancer risk. Collectively, our findings add to the growing body of evidence supporting an inverse association between higher dietary TAC intake and breast cancer risk. Second, all eligible articles were strictly screened according to predefined inclusion and exclusion criteria. Third, incident breast cancer cases were ascertained through medical records review, reducing the likelihood of misdiagnosis. Fourth, subgroup and sensitivity analyses further enhanced the robustness of our findings. Fifth, visual inspection of the funnel plot revealed little evidence of asymmetry, and statistical tests for publication bias were also non-significant, indicating a lower risk of publication bias. Finally, we conducted a dose-response analysis to further clarify the association between dietary TAC and breast cancer risk. Despite the aforementioned strengths, several important limitations should be taken into consideration. First, the observational nature of all included studies (mainly case-control studies) precluded us from drawing causality. Moreover, seven of the included studies were case-control studies, which are inherently susceptible to recall and selection bias. The predominance of case-control studies may inflate associations due to differential recall of dietary habits. Further longitudinal studies or randomized controlled trials are necessary to substantiate these findings. Second, reliance on self-reported FFQs or 24 h dietary recall to collect dietary data might introduce misclassification bias, resulting in the over or under-estimation of dietary TAC. Moreover, TAC databases are not globally harmonized. Most included studies relied on the USDA ORAC database, which is comprehensive for US foods but may not accurately reflect the antioxidant content of foods consumed in other countries. Thus, it is important to emphasize that dietary TAC estimation is inherently indirect and prone to error. Third, an important limitation is the potential for residual confounding, particularly healthy user bias. Higher TAC intake may be a proxy for overall healthy dietary patterns and lifestyle behaviors, including higher physical activity and socioeconomic status. While most included studies adjusted for major lifestyle factors, residual confounding due to unmeasured factors could not be completely excluded. Furthermore, our analysis focused on dietary TAC from foods rather than supplements; this approach reduces confounding from high-dose supplementation but may not capture the full antioxidant exposure spectrum. Fourth, substantial heterogeneity was observed in our meta-analysis. Although subgroup and sensitivity analyses were performed to explore possible sources of heterogeneity, we were unable to thoroughly analyze and explain the sources of inter-study heterogeneity. Fifth, the vast majority of eligible studies were performed in Iran, therefore, extrapolating our findings to other populations, especially Western countries, is premature. Further prospective studies in diverse populations are needed to confirm these findings. Finally, a possible selective reporting bias of included studies could not be ruled out, because we did not include gray literature in this meta-analysis.

## Conclusion

In conclusion, this meta-analysis showed that higher dietary TAC intake was inversely associated with breast cancer risk. Our findings further reinforce the existing evidence that higher dietary TAC was associated with a reduced risk of breast cancer risk. Nevertheless, the substantial heterogeneity observed across studies necessitates caution when interpreting these results. Further population-based longitudinal studies, particularly in different geographic regions and cultural backgrounds, are needed to confirm these findings.
