# Supplementary material for: Dietary total antioxidant capacity and risk of breast cancer: a systematic review and dose-response meta-analysis of observational studies
Source: Front Nutr. 2026 Aug 7;13:1890538. doi: 10.3389/fnut.2026.1890538 (PMC13495776; doi:10.3389/fnut.2026.1890538)
Supplement: Supplementary file 1 [file Table_1.doc]

**Search terms in PubMed, Embase, ISI Web of Science and Scopus**
(“dietary total antioxidant capacity”[all fields] OR “dietary antioxidant capacity” [all fields] OR “dietary TAC”[all fields] OR “dietary antioxidant index”[all fields] OR “non enzymatic antioxidant capacity”[all fields] OR “antioxidant capacity of diet”[all fields]) AND (“breast cancer”[all fields] OR “breast tumor”[all fields] OR “breast adenoma” [all fields] OR “breast neoplasms”[all fields] OR “breast carcinoma”[all fields])

**Search terms in China National Knowledge Infrastructure**

(膳食总抗氧化能力 OR 抗氧化剂) and 乳腺癌

**Search terms in other sources**

Hand-searching from reference lists of all relevant articles, previous reviews and meta-analyses was performed to identify relevant studies.
